# Pediatric Undifferentiated Pleomorphic Sarcoma of the Cecum

**DOI:** 10.31486/toj.22.0042

**Published:** 2023

**Authors:** Desiree McCombs, Kathleen Condon, Jessica Roybal, Rajasekharan Warrier, Corey Falcon

**Affiliations:** ^1^ The University of Queensland Medical School, Ochsner Clinical School, New Orleans, LA; ^2^ Department of Pediatrics, Tulane University School of Medicine, New Orleans, LA; ^3^ Department of Pediatric Surgery, Ochsner Hospital for Children, Ochsner Clinic Foundation, New Orleans, LA; ^4^ Department of Pediatric Hematology/Oncology, Ochsner Hospital for Children, Ochsner Clinic Foundation, New Orleans, LA

**Keywords:** *Colon–ascending*, *colonic neoplasms*, *gastrointestinal neoplasms*, *histiocytoma–malignant fibrous*, *sarcoma*

## Abstract

**Background:** Undifferentiated pleomorphic sarcoma (UPS) is a high-grade neoplasm typically diagnosed in older adults and localized to the extremities or retroperitoneum. Because of poor response to therapy and high rates of recurrence, this neoplasm is associated with a poor prognosis.

**Case Report:** A 12-year-old female presented with weight loss, abdominal pain, fatigue, and diarrhea. She was profoundly anemic with occult blood–positive stools. On endoscopy, a fungating cecal mass was biopsied and diagnosed as malignant sarcomatoid neoplasm. The neoplasm was resected with clear margins during subsequent surgery, and on final pathology was diagnosed as UPS. A suspicious lung nodule was also removed via video-assisted thoracoscopic surgery and found to be a granuloma positive for *Histoplasma capsulatum* for which the patient received antifungal therapy. The patient did not receive additional chemotherapy or radiotherapy and was doing well without signs of recurrence at 12 months postresection.

**Conclusion:** This report of cecal UPS in a 12-year-old is rare because of the patient's age and tumor location. We have identified only 2 other case reports of pediatric gastrointestinal UPS. This case illustrates the need for a broad differential and prompt workup in pediatric patients presenting with weight loss and abdominal complaints. More information regarding the management and outcomes in cases of gastrointestinal UPS is needed to assist providers in determining the best treatment course and to allow for better prognostication.

## INTRODUCTION

Undifferentiated pleomorphic sarcoma (UPS), formerly known as malignant fibrous histiocytoma (MFH), is a high-grade pleomorphic neoplasm without any definable line of differentiation.^1^ UPS usually occurs in the extremities or retroperitoneum; primary tumors of the gastrointestinal tract are uncommon. To our knowledge, only 14 cases of cecal or ascending colon UPS are reported in the literature.^2-14^ The classification and subdivision of these tumors went through several iterations until the World Health Organization 2002 classification eliminated the term MFH and replaced it with UPS.^1^

UPSs most commonly occur later in life, usually the sixth and seventh decades,^15^ and account for approximately 20% of all soft-tissue sarcomas.^8,16,17^ UPSs are more common in males than females with a 2.4:1 ratio.^7^

Risk factors for the development of UPS include genetics, radiation or chemotherapy exposure, chemical carcinogens, chronic postoperative repair, trauma, surgical incisions, and lymphedema.^7,18^ The cellular origins of UPSs are unclear, but they possibly arise from primitive mesenchymal stem cells that retain both fibroblastic and histiocytic potential and may present with markers and behaviors of both cell lines.^10,11^

A UPS typically presents as an enlarging lump that is often excised early when located on an extremity. However, intestinal UPSs are often discovered late when they have substantial tumor bulk. Presenting symptoms may include abdominal distention and pain, altered bowel habits, weight loss, anemia, blood in the stool, or palpable abdominal mass.^7,8^ Compared to other types of colon cancer, UPSs present more frequently with a right-sided mass, less frequently with constipation, and in up to 25% of cases patients report fevers.^7,8,10^ Laboratory findings at diagnosis may be normal or may show elevated inflammatory markers, leukocytosis, and anemia.^2,3,8,9^

Diagnosis is based on a combination of microscopic features and immunohistochemical staining techniques used to rule out other cell lines of origin. Lesions are typically characterized by pleomorphic, spindle-shaped cells with bizarre cytology and nuclear atypia.^19^ Immunohistochemical staining is sometimes positive for vimentin, actin, CD68, alpha 1-antitrypsin, alpha 1-antichymotrypsin, and laminin mRNA.^16,19^ Importantly, UPS has no reproducible immunophenotype or pattern of protein expression that allows for further classification of the tumor,^16^ and exclusion of pleomorphic variants of other neoplastic lines is required.^17^

Computed tomography (CT) typically shows a well-circumscribed and homogeneous mass or a low-density mass secondary to necrosis and hemorrhage.^19^ Masses are sometimes large and lobulated and may also have calcifications, hemorrhage, myxoid degeneration, necrosis, or tissue invasion.^16^

Standard treatment for UPS is early complete surgical resection with negative resection margins and en bloc lymph node dissection. The role of chemotherapy and radiation in the treatment of UPS is debated and without strong evidence.^8,10,11^ An increasing number of reports suggest that adjuvant chemotherapy or radiation may improve prognosis in certain clinical scenarios.^8,19^ Information regarding outcomes is primarily based on case reports or retrospective case series, and most reported cases of intestinal UPS were treated with surgery alone.^10,20^

The prognosis for UPS is generally poor because of regional invasiveness, distant metastases, and frequent recurrence.^7^ A review of 200 cases found a 2-year survival rate of 60%, a 5-year survival rate of 47%, and an overall recurrence rate of 44%. Metastasis occurred in 42% of cases, most commonly to the lungs (82%) but also to lymph nodes (32%).^15^

## CASE REPORT

A 12-year-old female presented to an urgent care clinic with 1 week of nausea and vomiting. She reported fatigue and chronic intermittent cramping abdominal pain for 4 months and unintentional weight loss of 25 pounds over the last 3 months. On examination, she had pale conjunctiva, tachycardia, and mild abdominal pain.

Initial complete blood count (CBC) revealed hemoglobin 7.3 g/dL (reference range, 12.0-16.0 g/dL), hematocrit 29.7% (reference range, 36.0%-46.0%), mean corpuscular volume 62 fL (reference range, 78.0-98.0 fL), red blood cell distribution width 18.9% (reference range, 11.5%-14.5%), and platelets 708 K/μL (reference range, 150-450 K/μL). Several days later, outpatient workup for anemia revealed serum iron of 10 μg/dL (reference range, 30-160 μg/dL), total iron binding capacity 304 μg/dL (reference range, 265-497 μg/dL), iron saturation 3% (reference range, 20%-50%), and ferritin 5 ng/mL (reference range, 16.0-300.0 ng/mL). The patient was found to be fecal occult blood–positive.

On outpatient recheck of CBC 6 days later, the patient's hemoglobin had dropped to 5.9 g/dL, and she was admitted to a community hospital where she received further workup and a blood transfusion. C-reactive protein (CRP) at admission was 3.01 mg/dL (reference range, 0.0-0.9 mg/dL). An initial concern was that the patient may have inflammatory bowel disease given her iron-deficiency anemia, elevated CRP, and positive fecal occult blood test with weight loss and diarrhea. Nonsteroidal anti-inflammatory drug (NSAID)–induced gastritis was also considered, given her daily NSAID use for abdominal pain. The patient was transferred the next day to a larger children's hospital to undergo further evaluation.

**Figure 1.**
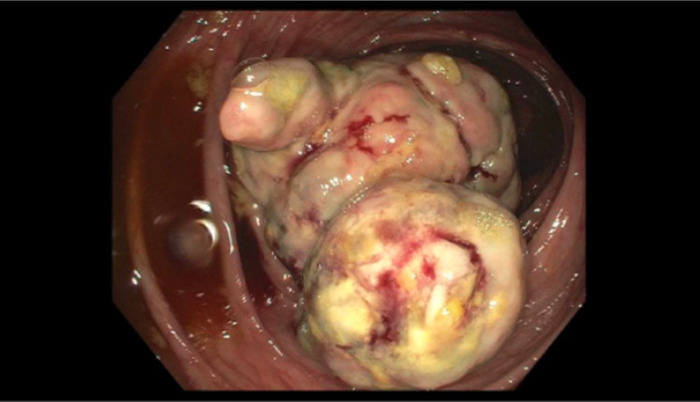
Colonoscopy revealed a fungating, nonobstructing, 7-cm cecal mass.

**Figure 2.**
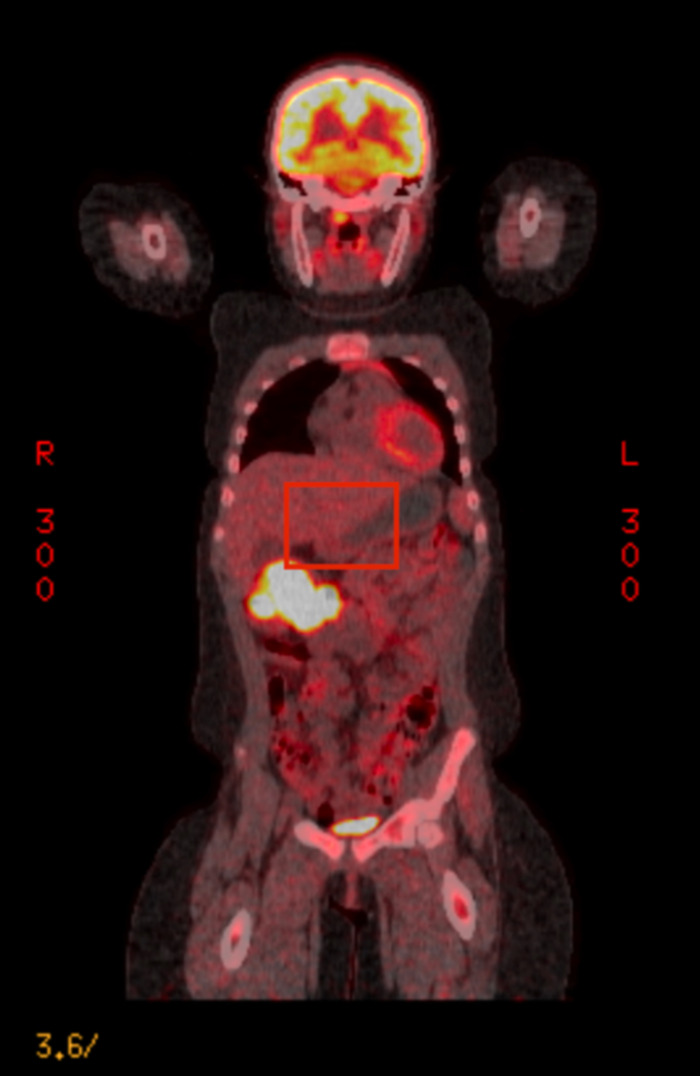
Positron emission tomography scan demonstrated notable increased uptake to the cecal mass in the right upper abdominal region.

Upper gastrointestinal endoscopy showed no abnormalities. Colonoscopy showed a large, fungating, nonobstructing, approximately 7-cm cecal mass (Figure 1). The remainder of the colon was normal. CT of the abdomen and pelvis noted a cecal mass and multiple enlarged pericolonic and mesenteric lymph nodes. CT of the chest with contrast revealed a solid noncalcified subpleural nodule (1.1 cm) in the posterior inferior left lower lobe. Positron emission tomography scan showed positive uptake in the right colon mass and possible uptake in the mediastinum and left lower lobe (Figure 2).

The mass was biopsied 18 days later, and the initial pathology diagnosis was malignant sarcomatoid neoplasm. Tumor cells were weakly positive for SATB2 and negative for keratin OSCAR, keratin AE1/AE3, desmin, myogenin, SMA, SOX10, S100, CD34, WT1, SALL4, and EMA.

**Figure 3.**
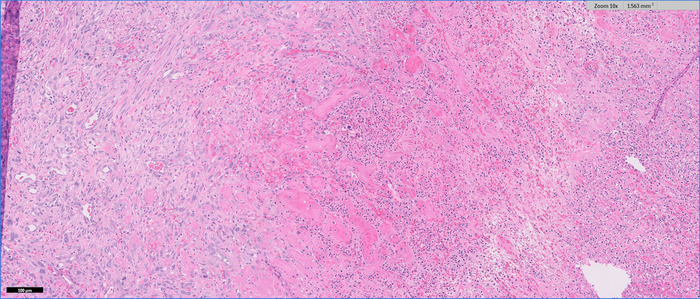
Histopathology of the resected specimen identified tumor cells on the left side and extensive necrosis on the right (magnification ×10).

**Figure 4.**
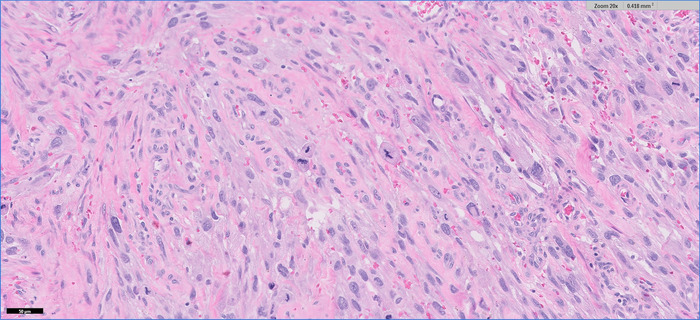
Histopathology of the resected specimen was notable for undifferentiated malignant tumor cells with focally pleomorphic forms and readily identified mitotic figures (magnification ×20).

A formal right hemicolectomy was performed without complication 6 days after the pathology results were received. At the time of surgery, an intraluminal cecal mass was noted to be causing colo-colonic intussusception. On final pathology 15 days following resection, the neoplasm was diagnosed as UPS (Figures 3 and 4). The ileal and colon margins were negative for malignancy. Thirty-nine regional lymph nodes were sampled and returned negative.

Ten days after the final pathology results were available and 25 days after colectomy, the patient underwent video-assisted thoracoscopic surgery with wedge resection of the pulmonary nodule. Pathology of the nodule 8 days later identified a 1.1-cm necrotizing granuloma. Small yeast with narrow-based budding without a mucicarmine-positive capsule was confirmed with Grocott methenamine silver and periodic acid-Schiff for fungus cytochemical stains. These findings were consistent with *Histoplasma capsulatum*, and the patient completed an 8-month course of itraconazole 300 mg daily. Chest x-rays after 6 months of treatment and several months after discontinuation of therapy were both negative for lung abnormalities concerning for recurrence.

Follow-up magnetic resonance imaging (MRI) of the abdomen at 2, 10, and 12 months postoperatively showed no evidence of disease. Multiple rechecks confirmed that the patient's anemia had resolved. Plans for long-term follow-up include annual endoscopy and MRI with biannual oncology visits for 5 years. After this period, the patient will be followed by oncology on an annual basis.

## DISCUSSION

UPS is a diagnosis of exclusion reserved for sarcomas with a distinct combination of immunohistochemical and microscopic features, made only after careful consideration of other differential diagnoses. This case is unique both for being localized to the gastrointestinal tract, with only 14 cases reported of cecal or ascending colon UPS, as well as the young age of the patient. To our knowledge, only 2 other cases of gastrointestinal UPS have been reported in the pediatric population.^13,21^

Our patient developed a nonobstructing right-sided colonic mass, with systemic features of disease including elevated inflammatory markers, anemia, and weight loss, which are consistent with the presentation of UPS. Despite the tumor's large size at diagnosis (7 cm), chemotherapy was not given in this case as the tumor was resected with clear margins and the patient had no lymph node involvement or metastatic disease. A lung lesion that was initially suspicious was resected and found to be positive for *Histoplasma capsulatum.* The patient will be followed by oncology to monitor for recurrence through periodic imaging and laboratory workup.

Additional research into the role of radiation and chemo-therapy for abdominal UPS is needed, especially for cases in which surgical resection is not possible. Continued monitoring and close follow-up are essential for a good long-term outcome because of the relatively high recurrence rate for UPS.

## CONCLUSION

Although malignancies of the gastrointestinal tract in pediatric patients are rare, this case exemplifies the need to maintain a broad differential. The case further shows the value of prompt referral and workup by appropriate medical specialists to limit morbidity and mortality. The treatment and long-term outcomes for pediatric UPS are an area with limited published literature that would benefit from further study of the long-term patient outcomes to guide clinical management.

## Notes

*This article meets the Accreditation Council for Graduate Medical Education and the American Board of Medical Specialties Maintenance of Certification competencies for Patient Care and Medical Knowledge.*

## References

[1] World Health Organization, International Agency for Research on Cancer. Pathology and Genetics of Tumours of Soft Tissue and Bone. Fletcher CDM, Unni KK, Mertens F, eds. IARC Press; 2002.

[2] Luna-Pérez P, Rodríguez DF, Luján L, Colorectal sarcoma: analysis of failure patterns. J Surg Oncol. 1998;69(1):36-40. doi: 10.1002/(sici)1096-9098(199809)69:1<36::aid-jso7>3.0.co;2-l 9762889

[3] Satake T, Matsuyama M. Cytologic features of ascites in malignant fibrous histiocytoma of the colon. Acta Pathol Jpn. 1988;38(7):921-928. doi: 10.1111/j.1440-1827.1988.tb02363.x 2847480

[4] Fukino S, Fukata T, Okano K, A case of malignant fibrous histiocytoma of the cecum. In Japanese. Nihon Geka Gakkai Zasshi. 1990;91(11):1752-1755. 2177519

[5] Murata I, Makiyama K, Miyazaki K, A case of inflammatory malignant fibrous histiocytoma of the colon. Gastroenterol Jpn. 1993;28(4):554-563. doi: 10.1007/BF02776955 7690726

[6] Hiraoka N, Mukai M, Suzuki M, Malignant fibrous histiocytoma of the cecum: report of a case and review of the literature. Pathol Int. 1997;47(10):718-724. doi: 10.1111/j.1440-1827.1997.tb04448.x 9361108

[7] Fu DL, Yang F, Maskay A, Primary intestinal malignant fibrous histiocytoma: two case reports. World J Gastroenterol. 2007;13(8):1299-1302. 17451221 10.3748/wjg.v13.i8.1299 PMC4147015

[8] Ji W, Zhong M, You Y, Hu KE, Wu B. Primary malignant fibrous histiocytoma of the colon: a case report and review of the literature. Mol Clin Oncol. 2016;4(6):1006-1008. doi: 10.3892/mco.2016.849 27284436 PMC4887843

[9] Du JF, Chen JW, Li F, Tian J, Xie X, Mao N. Concurrence of malignant fibrohistiocytoma and Takayasu arteritis: a case report. Rheumatol Int. 2012;32(10):3225-3227. doi: 10.1007/s00296-011-2156-8 21969061

[10] Okubo H, Ozeki K, Tanaka T, Matsuo T, Mochinaga N. Primary malignant fibrous histiocytoma of the ascending colon: report of a case. Surg Today. 2005;35(4):323-327. doi: 10.1007/s00595-004-2915-1 15815852

[11] Gupta C, Malani AK. Primary malignant fibrous histiocytoma of the colon. Clin Gastroenterol Hepatol. 2006;4(6):xxviii. doi: 10.1016/j.cgh.2006.01.004 16630767

[12] Udaka T, Suzuki Y, Kimura H, Miyashita K, Suwaki T, Yoshino T. Primary malignant fibrous histiocytoma of the ascending colon: report of a case. Surg Today. 1999;29(2):160-164. doi: 10.1007/BF02482242 10030742

[13] Huang Z, Wei K. Malignant fibrous histiocytoma of the ascending colon in a child. Am J Gastroenterol. 1993;88(6):972-973. 8389096

[14] Kazama S, Gokita T, Takano M, G-CSF-producing undifferentiated pleomorphic sarcoma adjacent to the ascending colon and in the right iliopsoas muscle: a case report and review of the literature. Intern Med. 2019;58(19):2783-2789. doi: 10.2169/internalmedicine.2762-19 31243197 PMC6815886

[15] Weiss SW, Enzinger FM. Malignant fibrous histiocytoma: an analysis of 200 cases. Cancer. 1978;41(6):2250-2266. doi: 10.1002/1097-0142(197806)41:6<2250::aid-cncr2820410626>3.0.co;2-w 207408

[16] Ziwei X, Yueming S. Recurrent adult intraabdominal undifferentiated high-grade pleomorphic sarcoma infiltrated the descending colon: a case report and review of the literature. J Gastrointest Cancer. 2019;50(3):629-633. doi: 10.1007/s12029-018-0071-x 29577178

[17] Diaz-Beveridge R, Melian M, Zac C, Primary mesenteric undifferentiated pleomorphic sarcoma masquerading as a colon carcinoma: a case report and review of the literature. Case Rep Oncol Med. 2015;2015:532656. doi: 10.1155/2015/532656 26380135 PMC4563063

[18] Azizi R, Mahjoubi B, Shayanfar N, Anaraki F, Zahedi-Shoolami L. Malignant fibrous histiocytoma of rectum: report of a case. Int J Surg Case Rep. 2011;2(6):111-113. doi: 10.1016/j.ijscr.2011.01.013 22096699 PMC3199686

[19] Lee JH, Kang DB, Park WC. Primary undifferentiated pleomorphic sarcoma of the colon mesentery. Ann Coloproctol. 2019;35(3):152-154. doi: 10.3393/ac.2018.03.11 31142106 PMC6625777

[20] Zagars GK, Mullen JR, Pollack A. Malignant fibrous histiocytoma: outcome and prognostic factors following conservation surgery and radiotherapy. Int J Radiat Oncol Biol Phys. 1996;34(5):983-994. doi: 10.1016/0360-3016(95)02262-7 8600111

[21] Carip C, de Beaumont T. Malignant histiocytofibroma of the small intestine in a young immune deficient patient. In French. Presse Med. 2002;31(5):214-216. 11878138

